# Contrast-enhanced ultrasound sonography optimises the assessment of lymph nodes in oncology

**DOI:** 10.3332/ecancer.2013.328

**Published:** 2013-06-26

**Authors:** Giuseppe Petralia, Giorgio Conte, Elvio De Fiori, Sarah Alessi, Massimo Bellomi

**Affiliations:** Department of Radiology, European Institute of Oncology, Milan 20121, Italy

**Keywords:** ultrasound, CEUS, lymph node, metastasis

## Abstract

Ultrasound sonography (US) plays an important role in the assessment of lymph nodes in oncology. However, ultrasound findings are often equivocal in not allowing the differentiation of reactive from metastatic lymph nodes. Here, we present the successful use of contrast-enhanced US in the assessment of a metastatic lymph node, improving the performance of conventional US and optimising the US-guided percutaneous biopsy.

## Image report

A 52-year-old patient who underwent a partial penectomy for a poorly differentiated squamous cell carcinoma (SCC) of the penis was followed up by ultrasound sonography (US) of the groin every four months.

**Figure 1:**
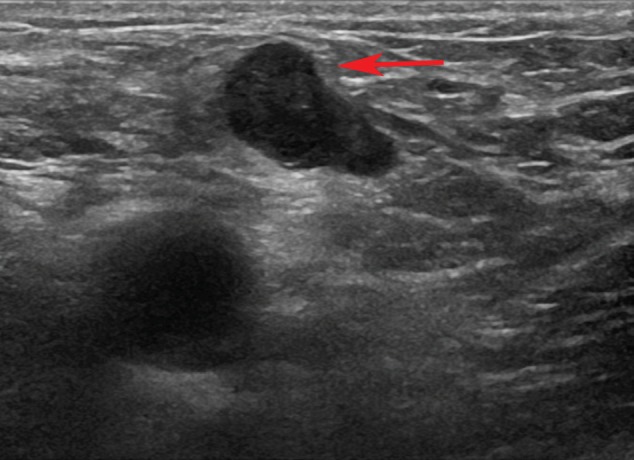
16-month follow-up US. The ultrasound exam shows a normal-sized lymph node in the right groin. It was considered suspicious because it has an asymmetric shape and cortical bulging (arrow).

**Figure 2:**
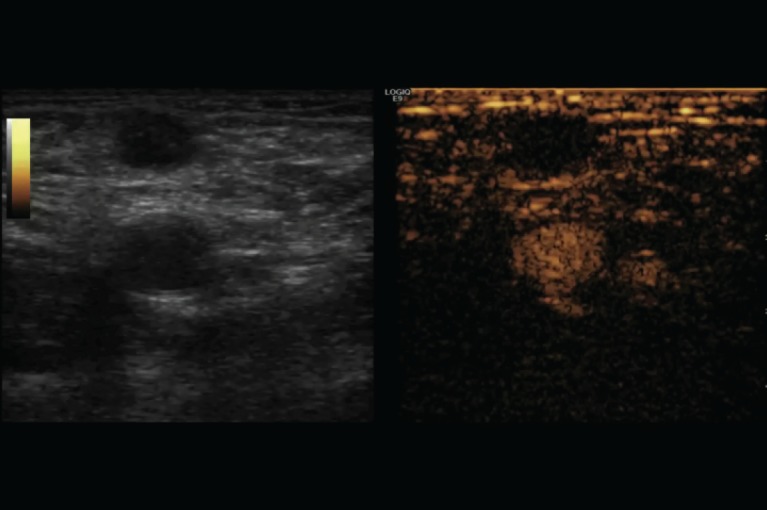

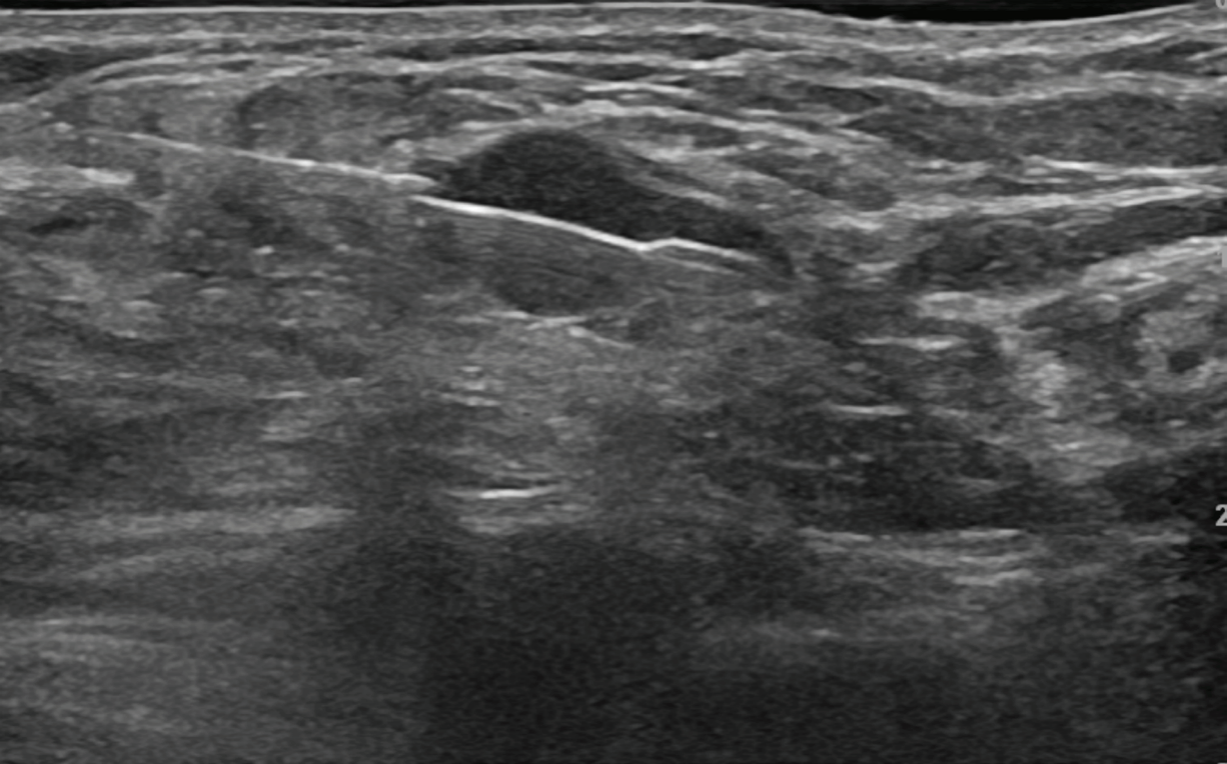
Figure 2: US-guided percutaneous biopsy of the suspicious lymph node. The US-guided percutaneous biopsy was performed using an 18-guage spring-loaded biopsy needle. Attention was paid to placing the notch of the stylet in correspondence to the hypoperfused area observed with CEUS.

**Figure 3:**
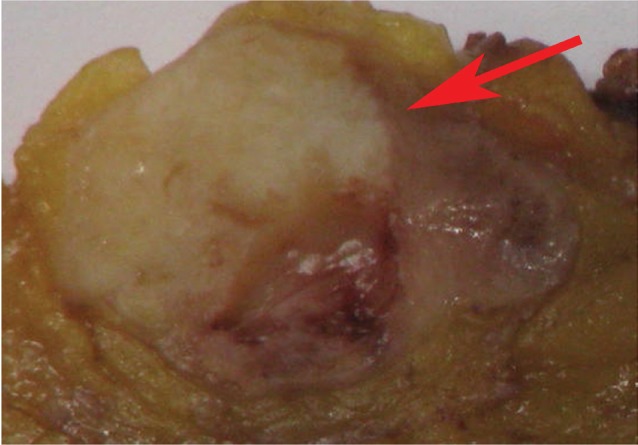
Histological evidence of metastatic deposit in the suspicious lymph node. Final histology confirmed the presence of a metastatic deposit (arrow) in the suspicious lymph node. Its shape visually matched the hypoperfused area visualised at CEUS.

**Figure 4:**
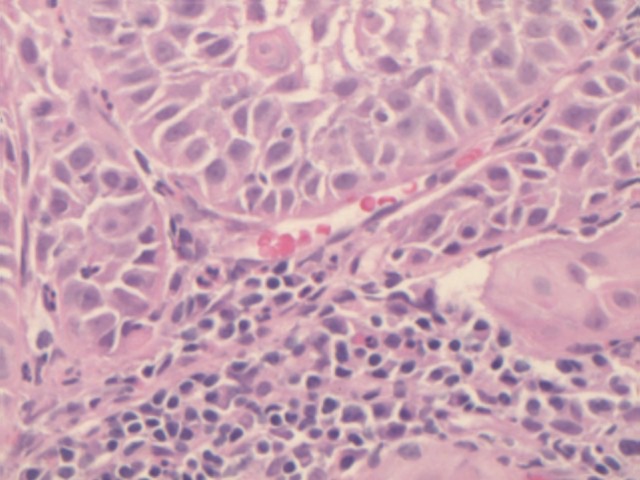
Histological examination. Haematoxylin and eosin histology (×20) showed cancerous squamous cells in the lymph node parenchyma.

At the 16-month follow-up US, a normal-sized lymph node (15 × 7 mm^2^) in the right groin was considered suspicious because of its asymmetric shape due to a cortical bulging (Figure 1), suggesting a metastatic deposit. Nevertheless, these findings were not sufficient to define the lymph node’s status (reactive or metastatic) with certainty. We then decided to perform a contrast-enhanced US (CEUS). A 3-mL bolus of contrast agent (sulphur-hexafluoride-filled microbubbles, Sonovue, Bracco International) was injected into a peripheral vein, followed by 10 mL of saline solution. A high-frequency probe (6–15 MHz) was placed on the right groin to visualise the largest area of the suspicious lymph node and was kept in a fixed position to visualise the dynamic phase, the CEUS exam (duration: 120 s). The lymph node was scanned in ‘contrast-tuned imaging technology’ mode, receiving the signal emitted by the microbubbles in a selective manner, thereby eliminating all signals that were not useful [1]. The arterial phase showed a nonenhancing focal area within the lymph node, accounting for a hypoperfused mass, surrounded by a fast enhancing normal lymph node parenchyma (Video). The hypoperfused focal area within the lymph node was considered suspicious for metastatic deposit. Therefore, a US-guided transcutaneous biopsy of the lymph node was performed, paying attention to sampling as much as possible of the hypoperfused focal area using an 18-gauge spring-loaded biopsy needle (Biopsy-bell, Mirandola, Italy) (Figure 2). The histological analysis of the sampled tissue revealed the presence of a metastatic deposit from SCC. The lymph node was then resected along with 27 other lymph nodes (superficial and deep right groin lymph nodes). The final histology confirmed the presence of a metastatic deposit in the suspicious lymph node (Figures 3 and 4), corresponding to the hypoperfused area visualised by CEUS.

In conclusion, CEUS improved the visualisation of a metastatic deposit within a suspicious lymph node and allowed a targeted biopsy of the suspicious area. Further experience would be recommended to validate this encouraging observation.

## Additional File



## Figures and Tables

**To view this video click here: http://ecancer.org/journal/7/full/328-Contrast-enhanced-ultrasound-sonography-optimises-the-assessment-of-lymphnodes-in-oncology.php**

**Video: The dynamic phases of the CEUS.**
